# Harnessing the power of regional baselines for broad‐scale genetic stock identification: A multistage, integrated, and cost‐effective approach

**DOI:** 10.1111/eva.13621

**Published:** 2023-11-30

**Authors:** Bobby Hsu, Christopher Habicht

**Affiliations:** ^1^ Commercial Fisheries Division Alaska Department of Fish and Game Anchorage Alaska USA

**Keywords:** Bayesian hierarchical modeling, coast‐wide genetic baseline, fishery management, genetic stock identification, mixed‐stock analysis, two‐step approach

## Abstract

In mixed‐stock fishery analyses, genetic stock identification (GSI) estimates the contribution of each population to a mixture and is typically conducted at a regional scale using genetic baselines specific to the stocks expected in that region. Often these regional baselines cannot be combined to produce broader geographical baselines due to non‐overlapping populations and genetic markers. In cases where the mixture contains stocks spanning across a wide area, a broad‐scale baseline is created, but often at the cost of resolution. Here, we introduce a new GSI method to harness the resolution capabilities of baselines developed for regional applications in the analysis of mixtures containing individuals from a broad geographic range. This method employs a multistage framework that allows disparate baselines to be used in a single integrated process that produces estimates along with the propagated errors from each stage. All individuals in the mixture sample are required to be genotyped for all genetic markers in the baselines used by this model, but the baselines do not require overlap in genetic markers or populations representing the broad‐scale or regional baselines. We demonstrate the utility of our integrated multistage model using a synthesized data set made up of Chinook salmon, *Oncorhynchus tshawytscha*, from the North Bering Sea of Alaska. The results show an improved accuracy for estimates using an integrated multistage framework, compared to the conventional framework of using separate hierarchical steps. The integrated multistage framework allows GSI of a wide geographic area without first developing a large scale, high‐resolution genetic baseline or dividing a mixture sample into smaller regions beforehand. This approach is more cost‐effective than updating range‐wide baselines with all regionally important markers.

## 1 INTRODUCTION

Genetic stock identification (GSI) is used to estimate the population composition of a sample from a fishery comprising of multiple stocks (i.e., a mixture) by comparing mixture genotypes to a baseline of allele frequency estimates from populations that represent all stocks, or reporting groups, that may be present in the mixture. This method requires the baseline to include allele frequency estimates for all the genetic markers for all the populations. Genetic baselines are often developed regionally, specifically designed to distinguish among local groups in regional mixed‐stock fisheries (e.g., Ackerman et al., 2011; Beacham et al., 2020, 2021; Ensing et al., 2013; Euclide et al., 2021; Flannery et al., 2010; Gilbey et al., 2018; Habicht et al., 2007; Seeb et al., 2000). Often these regional baselines cannot be combined easily across regions to produce broader geographical baselines because the type of genetic markers (e.g., microsatellites, single‐nucleotide polymorphisms) may not match or do not overlap at an adequate set of loci to differentiate reporting groups.

Traditionally, researchers from multiple laboratories have screened common sets of markers across populations representing larger geographic areas to conduct broad‐scale GSI analysis (e.g., Kondzela et al., 2002; Seeb et al., 2007; Teel et al., 2014; Templin et al., 2011), especially for high‐seas mixtures (e.g., Beacham et al., 2005, 2009; McKinney et al., 2020; Wirgin et al., 2020). These baselines require a high level of coordination among laboratories to standardize genetic markers designed to allocate fish to broad‐scale reporting groups, and they have been used successfully (Seeb et al., 2007; Stephenson et al., 2009). However, increasing resolution at the broad‐scale level can be expensive and logistically challenging because markers useful in some regions may provide no information in others and additional markers may be necessary to sufficiently resolve fine‐scale reporting groups. Incorporating new or additional genetic markers into a single broad‐scale baseline can require multiple laboratories to genotype their respective baseline populations or extensively share baseline tissues or DNA with the laboratory doing the baseline update (e.g., DeCovich et al., 2012). Obtaining tissues or DNA from the required coastwide populations can be difficult, especially if tissue samples are unavailable because they have been exhausted in previous analyses or are not shared by other researchers. Genotyping the large numbers of fish in broad‐scale baselines is also costly.

As an alternative to developing and using a comprehensive broad‐scale baseline, some researchers have adopted a hierarchical, “hard cut‐off” two‐step (HC 2‐step) GSI approach: fish are first individually assigned to broad‐scale reporting groups using established coastwide genetic baselines, then, fish assigned to certain reporting groups with probabilities surpassing a set threshold are genotyped for additional genetic markers and assigned to fine‐scale reporting groups using a regional baseline (e.g., Miller et al., 2010; Samarasin et al., 2019). However, in the HC 2‐step approach, these two GSI analyses are conducted independently such that individual fish might be misassigned in the first step, and the uncertainty incurred cannot be accounted for in the second step.

Here, we develop an integrated multistage GSI (Ms.GSI) model that combines the two‐step method into a single process. The Ms.GSI model is essentially two standard Bayesian GSI models (Pella‐Masuda model hereafter; Pella & Masuda, 2001) stacked on top of each other; hence the name “multistage.” Estimations of group memberships and population compositions in a Bayesian GSI model are done through iterations of the Markov Chain Monte Carlo algorithm called the Gibbs sampler (Casella & George, 1992). Note that the Gibbs sampler can be done using the standard, fully‐Bayesian or the conditional GSI model (Moran & Anderson, 2018).

We constructed the Ms.GSI framework by integrating both steps of the HC 2‐step method into each iteration of the Gibbs sampler process. The Ms.GSI workflow starts with a mixture with each fish genotyped for all the genetic markers in both the broad‐scale and regional baselines. During each iteration of the Gibbs sampler, at the first stage, each fish in the mixture is assigned a group membership from the populations in the broad‐scale baseline, and reporting group proportions are drawn from their full conditional distribution (i.e., the distribution of a parameter conditioned on the data and the current state of all other parameters and variables in the model) based on numbers of fish counted in each group. At the second stage, each fish in the mixture is assigned a group membership from the populations in the regional baseline; however, with only individuals assigned to broad‐scale populations that belong to areas represented by regional reporting groups, regional population proportions are drawn from a distribution based on numbers of fish counted in each of the regional population. This algorithm is repeated thousands of times until simulations of the Gibbs sampler converge to a stable state that is called the posterior distribution. The two stages are integrated as a single process that holistically captures errors incurred at both stages.

We demonstrate the Ms.GSI framework using a case study, with mixture data synthesized from existing genetic baselines using a cross‐validation analysis. That is, genotypes of fish are randomly selected from existing baselines to synthesize a mixture data set, and the fish selected for the mixture are excluded from the baseline data sets. By predetermining the group proportions of the simulated data set, we quantify the precision and accuracy of the estimates by comparing them to true values. Also, using the results of cross‐validation analysis, we compared Ms.GSI method to the conventional HC 2‐step approach.

## 2 METHODS

### 2.1 Model descriptions

#### 2.1.1 Single‐baseline models: Pella‐Masuda and conditional GSI

In this section, we first describe the single‐baseline Pella‐Masuda and the conditional GSI models before discussing the implementation of an integrated multistage framework.

In a group of mixed populations, the Pella‐Masuda model assigns group memberships to each individual based on its genetic make‐up (i.e., genotype). Then the model estimates overall population proportions by sampling from the distributions based on the numbers of individuals assigned to each population. In the fishery context, genetic data of the individuals are called the mixture because it consists of multi‐locus genotypes of individual fish collected from a mixed‐stock fishery. x denotes the mixture. Here, we use bold letters to represent a set of numbers, or a collection of distinct elements. For example, x is a set that contains individual x elements. $x_{m,l,j}$ is the count of allele j in locus l for individual fish m, where $m\in \left\{1,2,\ldots ,M\right\}$, $l\in \left\{1,2,\ldots ,L\right\}$, and $j\in \left\{1,2,\ldots ,J_{l}\right\}$ depends on locus l.

A baseline is the genetic data set of the known spawning populations and consists of allele counts of various reference populations collected at their spawning locations. Researchers select sampling locations to best represent demographic production and genetic diversity of populations in an area. y denotes the baseline. $y_{k,l,j}$ is the count of allele j in locus l for a sample of size $n_{k,l}$ collected from reference population k, where $k\in \left\{1,2,\ldots ,K\right\}$. It is assumed that y follow a multinomial distribution with parameters q deriving from the allele frequencies of reference populations. y is expressed as follows:

(1) $$y_{k}∼\text{Mult}\left(n_{k},q_{k}\right).$$

And a commonly used prior distribution for q is:

(2) $$q_{k}∼\text{Dirich}\left(\beta \right),$$

where $\beta =1/J_{l}$.

The distribution of allele counts of an individual should resemble the allele frequencies of the reference population which it came from. However, the group membership of the individual in the mixture is unknown so it needs to be estimated. Let $z_{m}$ represent the group membership for the *m*th mixture individual. $z_{m}$ is composed of 0's and a single 1 with a length of K (i.e., number of reference populations in the baseline). $z_{m,k}=1$ if individual m belongs to population k, and =0 otherwise.

We place a multinomial distribution on $z_{m,1},z_{m,2},\ldots ,z_{m,K}$ with size 1 and probabilities equal to population proportions $p_{1},p_{2},\ldots ,p_{K}$. We specify a Dirichlet prior distribution on $p_{1},p_{2},\ldots ,p_{K}$ with hyperparameters $\alpha_{1},\alpha_{2},\ldots ,\alpha_{K}$, where $\alpha_{1}=\alpha_{2}=\ldots =\alpha_{K}=1/K$. We express z as follows:

(3) $$z_{m}∼\text{Mult}\left(1,p\right),$$

with the prior distribution for p:

(4) $$p∼\text{Dirich}\left(\alpha \right),$$

where α=1/K.

For the mixture, allele counts in each locus of individuals also follow a multinomial distribution. The parameters are allele frequencies of the corresponding reference population with size equal to the ploidy of each respective locus. Note that group membership $z_{m,k}=1$ if individual m belongs to population k, and =0 otherwise. When multiplying (element‐wise) group memberships, $z_{m,1},z_{m,2},\ldots ,z_{m,K}$, and allele frequencies of reference populations, $q_{1},q_{2},\ldots ,q_{K}$, only allele frequencies of the reference population to which individual m belongs would remain while the rest go to zero. x is expressed as follows:

(5) $$x_{m}∼\text{Mult}\left(\text{ploidy},z_{m}\circ q\right),$$

where $\text{ploidy}={\text{ploidy}}_{1},{\text{ploidy}}_{2},\ldots ,{\text{ploidy}}_{L}$ denotes ploidy of each locus. We use ∘ to denote the element‐wise product.

Moran and Anderson (2018) implemented a genetic mixture analysis that utilized a model structure called the conditional GSI. The main difference between the Pella‐Masuda (i.e., fully Bayesian) and the conditional GSI models is that, in the conditional model, q is integrated out of the distribution of the mixture, x. That is, baseline allele frequencies are not being updated in the conditional model. The result is that the probability of x takes the form of a compound Dirichlet‐multinomial distribution (Johnson et al., 1997):

(6) $$x_{m}∼\mathrm{CDM}\left(\text{ploidy},z_{m}\circ v\right),$$

where v is β+y. Since q has been integrated out of $x_{m}$, the process for sampling parameters is simpler and more streamlined. We have implemented conditional GSI in each stage of the Ms.GSI model.

#### 2.1.2 Implementation of multistage

In a multistage setting, we refer to a baseline that covers the whole range of a mixed stock fishery as a broad‐scale baseline. The broad‐scale baseline typically covers a wide range of geographic areas but does not have a comprehensive collection of reference populations nor genetic markers to resolve differences between local populations within a sub‐region. These smaller sub‐regions of a broad‐scale baseline are covered by regional baselines with higher resolutions. We generalize the conditions of the Ms.GSI model to allow multiple regional baselines to be included, although we mainly deal with one regional baseline versus one broad‐scale baseline in this manuscript.

Let there be B populations in the broad‐scale baseline and indexed as b=1,2,…,B. Each of these broad‐scale populations may belong to exactly 0 or 1 sub‐region for which regional baselines might be available. These regional baselines have different sets of genetic markers than the broad‐scale baseline and typically include additional populations that are not represented in the broad‐scale baseline. Let there be R disjoint sub‐regions indexed by r, with each sub‐region represented by a distinctive regional baseline. We employ a superscript ^(*r*)^ upon variables to indicate a quantity associated with regional baseline r. Populations in different sub‐regions cannot overlap, and each population only occurs once among the regional baselines. Let k index the populations within these R regional baselines and $K_{r}$ denotes the number of populations within regional baseline r.

In the Ms.GSI framework, the two stages are connected because the regional group membership of an individual is conditional on whether the broad‐scale group membership of that individual belongs to the area of that particular sub‐region. The following describes the conditional relationship between the broad‐scale and the regional baselines:

(7) $$z_{m}^{\left(r\right)}|z_{m},B^{\left(r\right)}=\left\{\begin{array}{ll} z_{m}^{\left(r\right)} & \text{if}\;\sum_{b\in B^{\left(r\right)}}z_{m,b}=1,\\ 0 & \text{otherwise} \end{array}\right.$$

where $z_{m}$ and $z_{m}^{\left(r\right)}$ are vectors of indicators (0 or 1) identifying the broad‐scale and regional populations that individual m belongs to. $B^{\left(r\right)}$ denotes the broad‐scale populations that belong to the areas represented by the reporting groups of region r, and 0 is a vector of all zeros.

Ultimately, we want to estimate the fraction of individuals in the mixture that come from each of the sub‐regional populations, as well as from any of the populations in the broad‐scale baseline that are not associated with a regional baseline. $p_{k}^{\left(r\right)}$ denotes the mixture proportion of the *k*th population in region r, and $p_{b}$ denotes the mixture proportion of population b in the broad‐scale baseline. Thus, we endeavor to estimate the mixture proportions $p_{k}^{\left(r\right)}$ for each $\left(r,k\right)$ such that r=1,2,…,R and $k=1,2,\ldots ,K_{r}$ along with $p_{b}$, where $b\in B_{*}$, with $B_{*}$ denoting the broad‐scale populations that do not belong to any areas represented by regional baselines. Lastly, we multiply $p^{\left(r\right)}$ by $\sum_{b\in B^{\left(r\right)}}p_{b}$ for each region r, so the scaled $p^{\left(r\right)}$ for all regions and $p_{b}$, where $b\in B_{*}$ would sum to one.

#### 2.1.3 Gibbs sampler

Pella and Masuda (2001) and Moran and Anderson (2018) described the methods for the Pella‐Masuda and the conditional GSI model. We modified the methods and developed a Gibbs sampler algorithm for the Ms.GSI framework. We programmed the Gibbs sampler in *R* (R Core Team, 2022), and the program is available freely as an *R* package, Ms.GSI, at https://github.com/boppingshoe/Ms.GSI.

Conceptually, we use a Gibbs sampler to sample from the marginal posteriors of q, p, and z for the broad‐scale baseline and $q^{\left(r\right)}$, $p^{\left(r\right)}$, and $z^{\left(r\right)}$ for each sub‐regional baseline. Pella and Masuda (2001) derived the full conditional distributions for the Gibbs sampler in the context of a single‐baseline setting, and we modified them for the Ms.GSI framework.

The full conditional distributions for the Gibbs sampler at the broad‐scale stage are:

(8) $$q_{k,l}|x,y,z,\beta ∼\text{Dirich}\left(\sum_{m=1}^{M}x_{m,l,j}z_{m,b}+y_{b,l,j}+\beta_{l,j}\right),$$

(9) $$p|z,\alpha ∼\text{Dirich}\left(\sum_{m=1}^{M}z_{m,b}+\alpha_{b}\right),$$

(10) $$\text{and}\;z_{m}|p,q,x_{m}∼\text{Mult}\left(1,w_{m}\right),$$

(11) $$\text{where}\;w_{m,b}\propto p_{b}\cdot \prod_{l=1}^{L}\prod_{j=1}^{J_{l}}q_{b,l,j}^{x_{m,l,j}}.$$

The full conditional distributions for the Gibbs sampler at the regional stage are:

(12) $$q_{k,l}^{\left(r\right)}|x^{\left(r\right)},y^{\left(r\right)},z^{\left(r\right)},\beta^{\left(r\right)}∼\text{Dirich}\left(\sum_{m=1}^{M}x_{m,l,j}^{\left(r\right)}z_{m,k}^{\left(r\right)}+y_{k,l,j}^{\left(r\right)}+\beta_{l,j}^{\left(r\right)}\right),$$

(13) $$p^{\left(r\right)}|z^{\left(r\right)},z,B^{\left(r\right)},\alpha^{\left(r\right)}∼\text{Dirich}\left(\sum_{m=1}^{M}\left(z_{m,k}^{\left(r\right)}|z_{m,b},B_{m,b}^{\left(r\right)}\right)+\alpha_{k}^{\left(r\right)}\right),$$

(14) $${\text{and}\;z}_{m}^{\left(r\right)}|p^{\left(r\right)},q^{\left(r\right)},x_{m}^{\left(r\right)}∼\text{Mult}\left(1,w_{m}^{\left(r\right)}\right),$$

(15) $${\text{where}\;w}_{m,k}^{\left(r\right)}\propto \left(p_{k}^{\left(r\right)}\cdot \prod_{l=1}^{L}\prod_{j=1}^{J_{l}}{q^{\left(r\right)}}_{k,l,j}^{x_{m,l,j}}\right)$$

To initiate the Gibbs sampler for the Ms.GSI model, we begin the process by drawing starting values, $p^{\left(0\right)}$, $q^{\left(0\right)}$, ${p^{\left(r\right)}}^{\left(0\right)}$ and ${q^{\left(r\right)}}^{\left(0\right)}$, from their prior distributions. We use superscript ^(*t*)^ to denote *t*th iteration of the Gibbs sampler. Sampling for the fully Bayesian model proceeds as follows:

For t=1,2,…,T, repeat

1. Determine the group memberships of mixture individuals at the broad‐scale stage, $z_{m}^{\left(t\right)}|p^{\left(t-1\right)},q^{\left(t-1\right)},x_{m}∼\text{Mult}\left(1,w_{m}\right)$.
2. Determine the group memberships of mixture individuals for each sub‐region at the regional stage, ${z_{m}^{\left(r\right)}}^{\left(t\right)}|{p^{\left(r\right)}}^{\left(t-1\right)},{q^{\left(r\right)}}^{\left(t-1\right)},x_{m}^{\left(r\right)}∼\text{Mult}\left(1,w_{m}^{\left(r\right)}\right),r=1,2,\ldots ,R$.

Draw updated values, $q^{\left(t\right)}$, $p^{\left(t\right)}$, ${q^{\left(r\right)}}^{\left(t\right)}$, and ${p^{\left(r\right)}}^{\left(t\right)}$ from $p\left(q|x,y,z^{\left(t\right)},\beta \right)$, $p\left(p|z^{\left(t\right)},\alpha \right)$, $p\left(q^{\left(r\right)}|x^{\left(r\right)},y^{\left(r\right)},{z^{\left(r\right)}}^{\left(t\right)},\beta^{\left(r\right)}\right)$, and $p\left(p^{\left(r\right)}|{z^{\left(r\right)}}^{\left(t\right)},z^{\left(t\right)},B^{\left(r\right)},\alpha^{\left(r\right)}\right)$, respectively.

T should be large enough to ensure the simulations converge to the posterior distribution. Usually, it takes thousands of iterations. Implementing the conditional GSI model only requires a slight modification from the above algorithm. q and $q^{\left(r\right)}$ only need to be calculated once at the initial step without further updates, otherwise the procedures remain the same.

**FIGURE 1:**
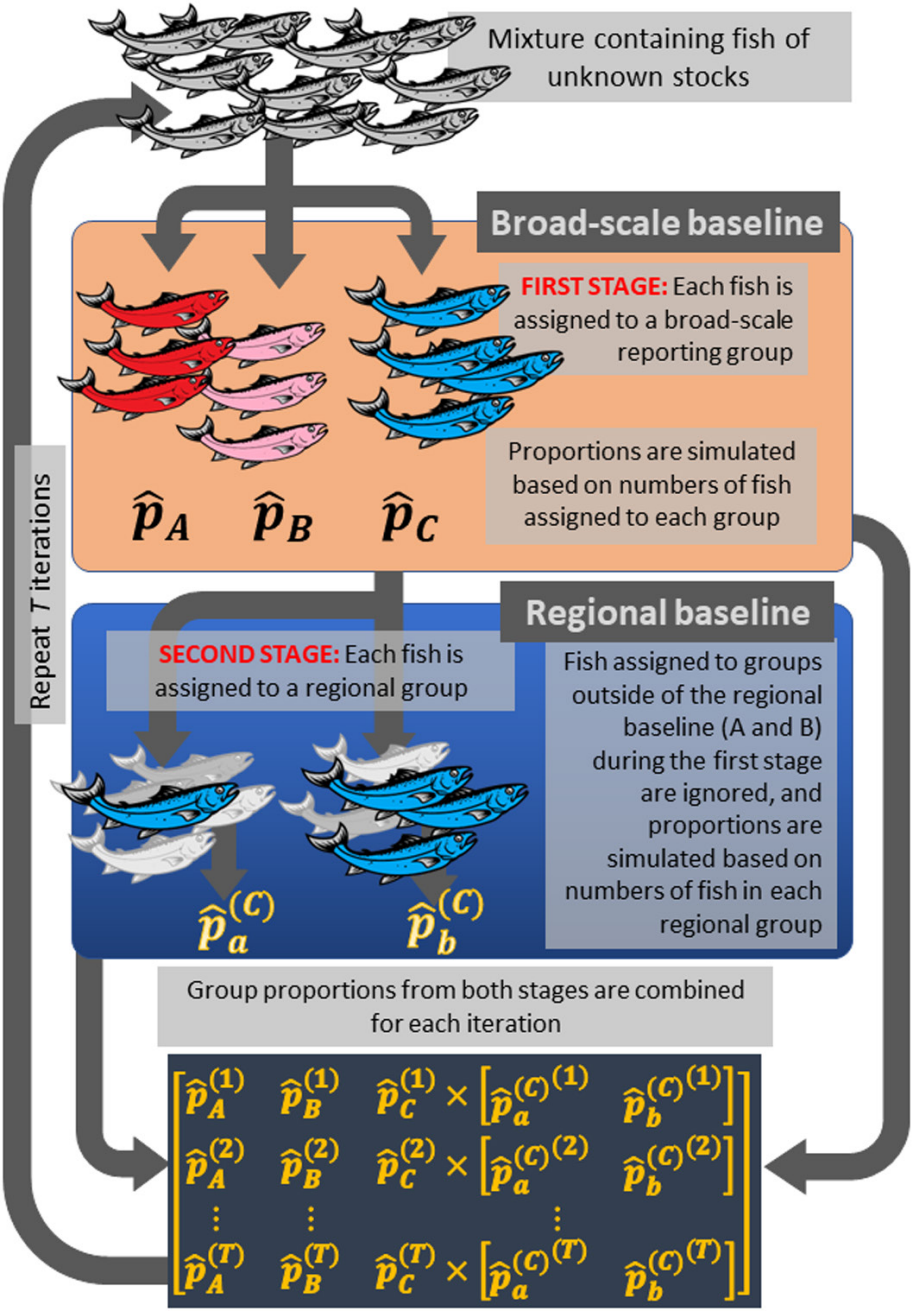
Summary of workflows for genetic stock identification in an integrated multistage framework with one broad‐scale and one regional baseline at each stage. Steps are repeated *T* iterations, and *T* should be large enough so that the simulations converge to the posterior distribution. Reporting group proportions estimated at each stage were combined and recorded during each iteration. At the first stage, the broad‐scale reporting groups were A, B, and C with respective proportions $p_{A}$, $p_{B}$, and $p_{C}$. Using a regional baseline, C was further divided into regional reporting groups a and b at the second stage with respective proportions $p_{a}^{\left(C\right)}$ and $p_{b}^{\left(C\right)}$. We combined the group proportions from the two stages as follows: $p_{A}$, $p_{B}$, $p_{C}\cdot \left(p_{a}^{\left(C\right)},p_{b}^{\left(C\right)}\right)$.

We also illustrate the Ms.GSI workflows in Figure 1.

### 2.2 Case study

We demonstrated the utility of the Ms.GSI model in a cross‐validation study in which we compared the Ms.GSI model to the HC 2‐step method along with the true proportions of a simulated mixture. In a hypothetical scenario, we created mixtures consisting of fish from coastal western Alaska (Coastal W. Alaska) and the Yukon River. By choosing from among currently available genetic markers, we created a broad‐scale baseline that had sufficient coverage for the mixture and was capable of resolving fish from Coastal W. Alaska versus the middle and upper Yukon River, but was purposely designed to be inadequate to resolve fish within the middle and upper Yukon River. For the populations from the middle and upper Yukon River, we included a regional baseline that was capable of distinguishing the local populations.

**FIGURE 2:**
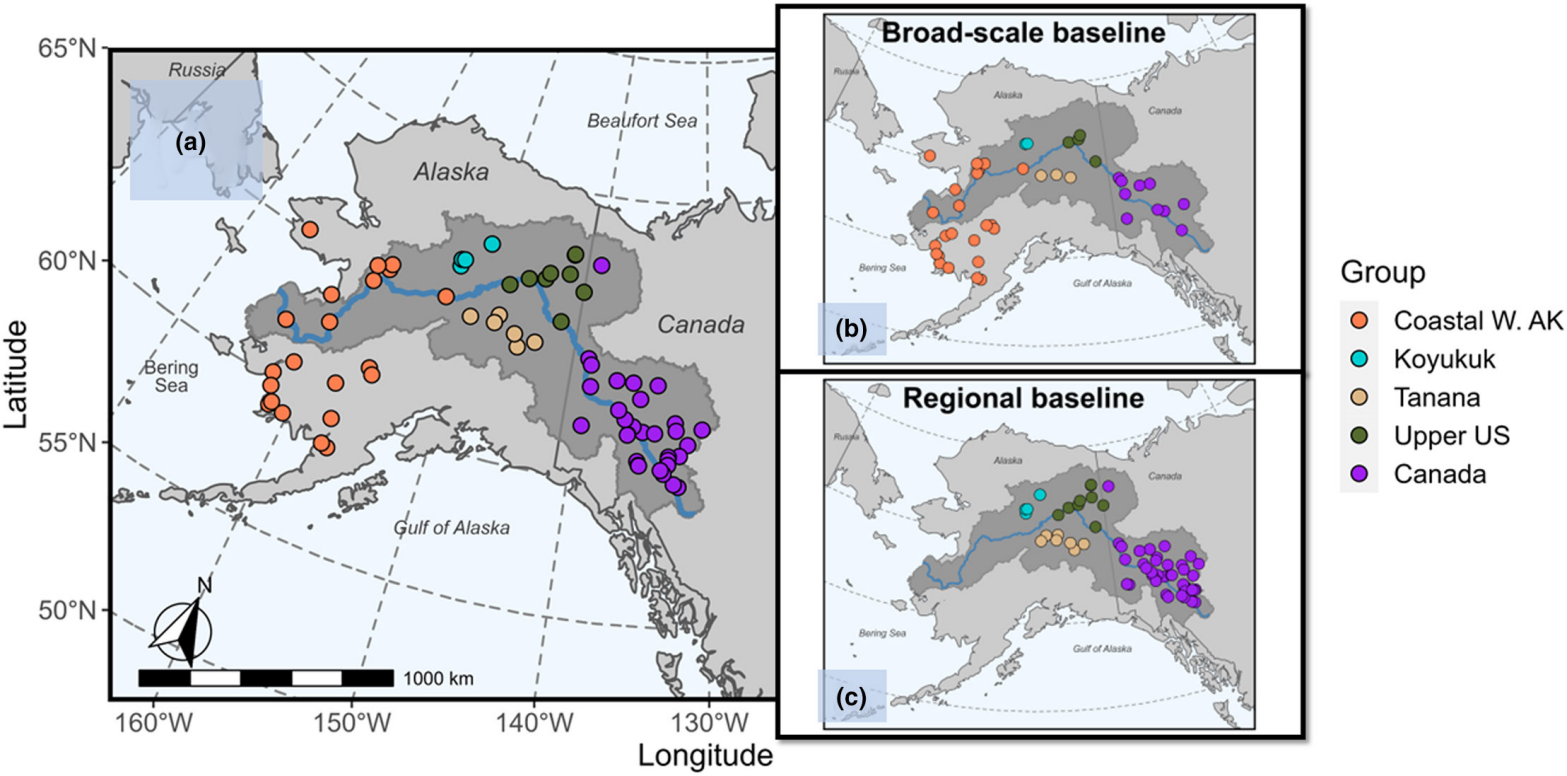
Map showing the collection locations in the North Bering Sea case study for (a) the simulated mixture sample, (b) the broad‐scale baseline (10 markers), and (c) the regional baseline (380 markers). Fill color represents reporting groups of Chinook salmon populations. Shaded area represents the Yukon River drainage.

We assembled a simulated mixture from existing Chinook salmon (*Oncorhynchus tshawytscha*) baselines for the Northern Bering Sea, Alaska. The DNA samples were collected during various projects in the past, and their genotype data were archived in a database maintained by the Alaska Department of Fish & Game Gene Conservation Lab (GCL). First, we retrieved genotypes from the GCL database from fish collected at 151 spawning sites for populations across Yukon River and Coastal W. Alaska (Figure 2a). This baseline had a marker‐set of 380 loci that could effectively differentiate Chinook salmon among four reporting groups from the middle/upper Yukon River (Koyukuk, Tanana, Upper U.S., and Canada; Lee et al., 2021; Figure 2c). Then, we randomly drew an arbitrary number of 205 fish from populations from five reporting groups from the coastal western region of Alaska and the Yukon River (Figure 2a) in proportions shown in Table 1. These proportions were unrelated to the true abundance in the actual fisheries.

**TABLE 1:** Numbers of populations, mixture proportion, sample size, and whether each reporting group in the broad‐scale baseline had a regional baseline.

Reporting group	*N* pop.	Mixture prop.	*N* samples	Regional
Coastal W. Alaska	41	0.27	55	No
Koyukuk	2	0.10	20	Yes
Tanana	3	0.19	39	Yes
Upper US	2	0.13	27	Yes
Canada	12	0.31	64	Yes

*Note*: “Mixture prop.” and “*N* samples” are the true proportion and sample size for each reporting group.

For the broad‐scale baseline, we used a baseline for the Northern Bering Sea juvenile Chinook salmon (84 loci and 60 populations collected from 111 areas; Howard et al., 2019; Table 2; Figure 2b). We included only a subset of the genetic markers screened in order to simulate a depauperate baseline that was capable of distinguishing among broad‐scale reporting groups, but not the fine‐scale reporting groups. We reduced the broad‐scale baseline data set to include only 10 of these genetic markers for all populations, which was adequate to distinguish two broad‐scale regions (Coastal W. Alaska vs. middle/upper Yukon River reporting groups). We selected these 10 loci also out of convenience because they were a subset of the 380 marker‐set described in the previous paragraph. It was not a requirement for the Ms.GSI and HC 2‐step models to have overlapping loci between the broad‐scale and regional baselines.

**TABLE 2:** Numbers of populations in each reporting unit for the regional baseline.

Reporting group	*N* pop.
Koyukuk	4
Tanana	5
Upper US	5
Canada	29

For the regional baseline, we used the full set of 380 genetic markers, but pared down the populations to only those representing the middle/upper Yukon River reporting groups (43 populations collected from 106 areas; Table 2; Figure 2c). This baseline was able to distinguish among the fine‐scale reporting groups in this area. Fish selected for the mixture data set were excluded from both baseline data sets.

#### 2.2.1 Ms.GSI

We ran the Gibbs sampler for the Ms.GSI model using our *R* package, Ms.GSI. To run the Gibbs sampler, we set an equal prior probability for all reporting groups (i.e., a flat prior) for each baseline. We ran the sampler in five independent chains, each with 25,000 iterations. We discarded the first 15,000 iterations of each chain as warm‐up runs (i.e., burn‐ins) and thinned the resulting posterior distribution by keeping every fifth sequence. We checked for convergence of the estimates among chains using Gelman‐Rubin diagnostics ($\hat{R}$ close to 1) and effective size (*N*_eff_ > 1000; Gelman et al., 2014; Gelman & Rubin, 1992) and by visually inspecting the trace plots for ample mixing between chains (Brooks & Gelman, 1998).

#### 2.2.2 HC 2‐step

We also conducted a GSI analysis on the same data set using a conventional HC 2‐step approach. We first assigned each fish in the data set to a broad‐scale reporting group. Fish that were assigned to a broad‐scale population that belonged to areas represented by regional reporting groups with a probability greater than 0.8 were selected for the second step. We used the regional baseline for the second GSI analysis. We conducted GSI for each baseline separately using the Gibbs sampler script we developed in *R* and followed the same procedures for numbers of MCMC chain, iterations, burn‐ins, and thinning as the Ms.GSI model.

#### 2.2.3 Model comparisons

We compared the results of Ms.GSI and the HC 2‐step approach to the true proportions of the synthesized data. We summarized and graphically inspected the membership assignment probabilities at the regional reporting groups level.

To quantify the performance of the models, we followed the procedures for genetic baseline evaluation outlined in Barclay et al. (2019) with modifications. Specifically, we repeated the cross‐validation analysis 50 times, each time with randomly chosen group proportions for the synthesized mixture. Then, we compared the mean estimates and the true values of the group proportions for each of the 50 analyses. The guidelines in Barclay et al. (2019) suggested that an adequate baseline should produce estimates falling within ±0.1 of the true values more than 90% of the time, with biases ≤±0.05, and have root‐mean‐square errors (RMSE) ≤0.05.

To measure how often the estimates deviated from the true values, we calculated the portion of mean estimates that were allocated beyond ±0.1 of the true values of the mixture proportions. Furthermore, we calculated bias and RMSE of the mean estimates for each model. We estimated bias by taking the difference between the mean of mean estimates and the mean of the true values. Root‐mean‐square error quantified bias and precision, and a smaller value for RMSE indicated less bias and higher precision. It was calculated as $\text{RMSE}=\sqrt{\frac{1}{n}\sum_{i=1}^{n}{\left(Y_{i}-{\hat{Y}}_{i}\right)}^{2}}$, where n was the number of analyses, $Y_{i}$ was the true proportion of group y for the *i*th iteration, and ${\hat{Y}}_{i}$ was the mean estimated proportion of group y for the *i*th iteration.

The 50 cross‐validation assessments also served as an evaluation to assess the adequacy of the broad‐scale baseline we selected for our analysis. Because we decreased the resolution of the broad‐scale baseline with a reduced marker‐set, we made sure that the reduced baseline could adequately distinguish the broad‐scale reporting groups chosen for our analysis.

To shorten computing time for the evaluation, we ran each of the 50 cross‐validation analyses with five chains of 5000 iterations instead of 25,000. We discarded the first half of each chain as a warm‐up run and did not thin the posterior output. Five thousands Gibbs sampler iterations would be adequate for this evaluation because we only compared posterior means to the true values. If we were to estimate intervals, we would run the Gibbs sampler with more iterations.

## 3 RESULTS

### 3.1 Case study

#### 3.1.1 Ms.GSI

For the single cross‐validation analysis, $\hat{R}$'s were all close to 1.0 and *N*_eff_'s were greater than 1000, which indicated no major concerns for the Gibbs sampler simulations of the Ms.GSI model (Table 3). Trace plots showed well mixed MCMC chains (plots not shown).

**TABLE 3:** Ms.GSI model estimates with convergence diagnostics for the Northern Bering Sea Chinook cross‐validation analysis.

Reporting group	True prop.	Mean	SD	Lower 90% CrI	Upper 90% CrI	$\hat{R}$	*N*_eff_
Coastal W. Alaska	0.27	0.29	0.05	0.21	0.37	1.00	1025.04
Koyukuk	0.10	0.08	0.02	0.05	0.13	1.00	4057.26
Tanana	0.19	0.20	0.03	0.14	0.26	1.00	2552.64
Upper US	0.13	0.13	0.03	0.09	0.18	1.00	6879.51
Canada	0.31	0.30	0.03	0.25	0.35	1.00	10,051.76

*Note*: “Mean,” “SD,” “Lower 90% CrI,” “Upper 90% CrI,” are the summary statistics for the marginal posterior distributions of each reporting group. $\hat{R}$ represents Gelman‐Rubin potential scale reduction factor. *N*_eff_ represents the effective size of posterior samples.

#### 3.1.2 HC 2‐step

For the single cross‐validation analysis, $\hat{R}$'s were all close to 1.0 and *N*_eff_'s were greater than 1000, which indicated no major concerns for the Gibbs sampler simulations of the HC 2‐step approach (Table 4). Trace plots showed well mixed MCMC chains (plots not shown).

**TABLE 4:** Combining estimates with convergence diagnostics for both steps of the HC 2‐step approach for the Northern Bering Sea Chinook cross‐validation analysis.

Reporting group	True prop.	Mean	SD	Lower 90% CrI	Upper 90% CrI	$\hat{R}$	*N*_eff_
Coastal W. Alaska	0.27	0.29	0.05	0.21	0.37	1.00	1423.89
Koyukuk	0.10	0.06	0.02	0.04	0.10	1.00	10127.19
Tanana	0.19	0.17	0.03	0.13	0.22	1.00	10,000.00
Upper US	0.13	0.13	0.02	0.09	0.17	1.00	9885.35
Canada	0.31	0.35	0.03	0.29	0.40	1.00	9772.68

*Note*: “Mean,” “SD,” “Lower 90% CrI,” “Upper 90% CrI,” are the summary statistics for the marginal posterior distributions of each reporting group. $\hat{R}$ represents Gelman‐Rubin potential scale reduction factor. *N*_eff_ represents the effective size of posterior samples. The results for “Coastal W. Alaska” are from the first step, and the results for “Koyukuk,” “Tanana,” “Upper US,” and “Canada” are from the second step of the 2‐step approach.

#### 3.1.3 Model comparisons

Baseline evaluation showed that the reduced broad‐scale baseline had an acceptable resolution in identifying broad‐scale reporting groups, albeit RMSE was slightly above the guideline of 0.05 (Table 5).

**TABLE 5:** Summary of model evaluation based on 50 cross‐validation analyses.

Group	RMSE (Ms.GSI)	RMSE (HC 2‐step)	Bias (Ms.GSI)	Bias (HC 2‐step)	% deviation (Ms.GSI)	% deviation (HC 2‐step)
Coastal W. Alaska	0.053	0.053	0.026	0.026	4	4
Koyukuk	0.041	0.094	−0.024	−0.026	4	26
Tanana	0.026	0.036	0.009	0.044	0	0
Upper US	0.016	0.044	−0.004	0.076	0	4
Canada	0.012	0.067	−0.007	0.110	0	10

*Note*: Root‐mean‐square errors (RMSE) measure the square root of average square errors of the reporting group proportions. Bias is the difference between the mean of mean estimates and the mean of the true values. “% Deviation” summarizes 50 mean estimates of each model that deviate more than ±0.1 from the true values for each reporting group.

**FIGURE 3:**
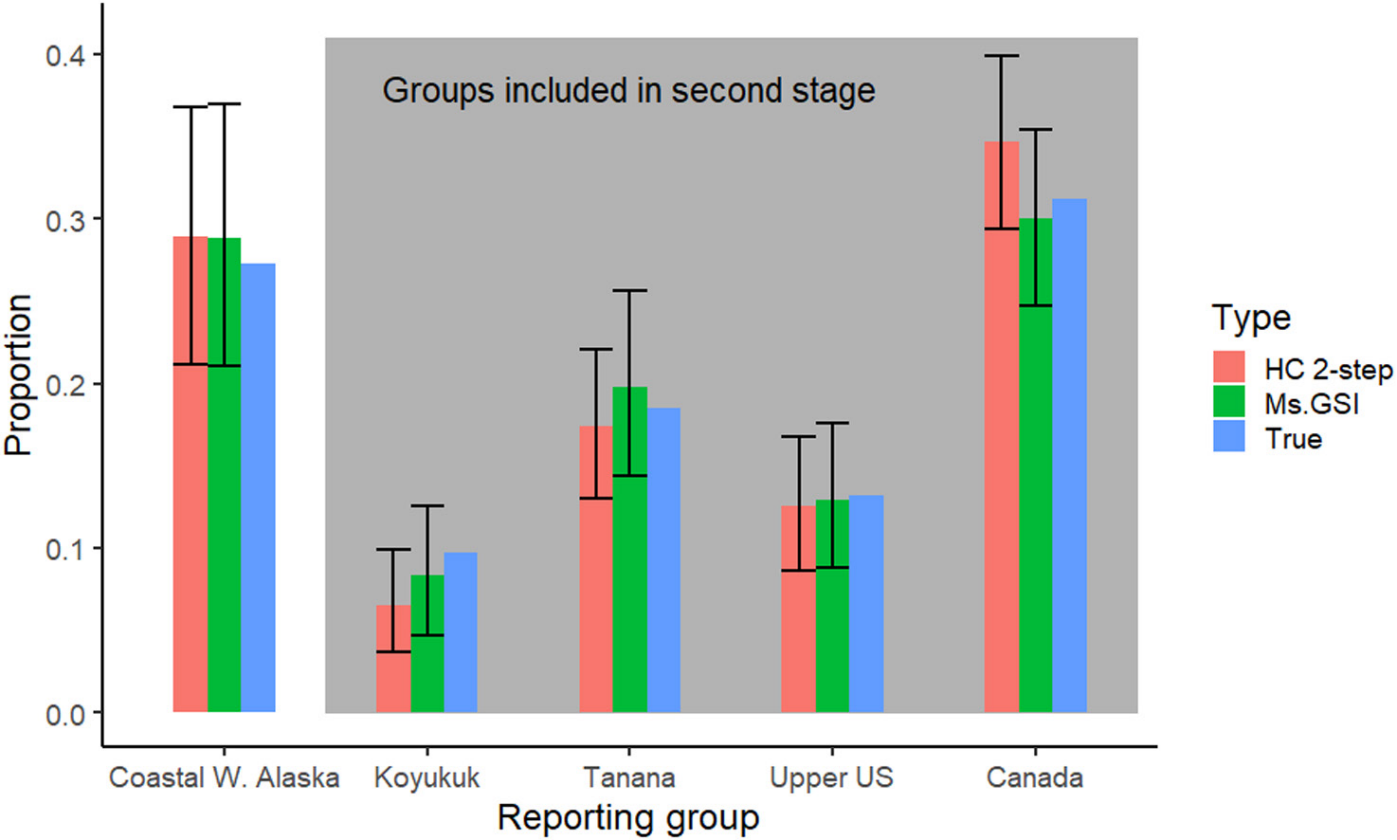
Comparison between estimates of the integrated multistage framework (Ms.GSI), the hard cut‐off two‐step approach (HC 2‐step), and true proportions of the five reporting groups in the cross‐validation analysis for Northern Bering Sea Chinook salmon. The width of credible intervals for the HC 2‐step approach are biased low because they do not include uncertainty of the first stage analysis (broad‐scale level).

Estimates from Ms.GSI were more accurate compared to estimates from the HC 2‐step approach (Table 5; Figure 3), especially for Koyukuk and Canada reporting groups. HC 2‐step approach underestimated the proportion of Koyukuk while it overestimated the proportion of the Canada reporting group. Integration of the two stages in Ms.GSI was able to correct the discrepancies found in the group proportions of HC 2‐step approach. There were no discernible differences in the estimates of Coastal W. Alaska between Ms.GSI and HC 2‐step because Coastal W. Alaska was estimated using only the broad‐scale baseline in both frameworks.

Ms.GSI showed slightly wider credible intervals for its estimates of the regional groups compared to the HC 2‐step approach (Figure 3). There were wider intervals for the Ms.GSI model because it accounted for uncertainties incurred at both stages while the HC 2‐step approach did not account for uncertainties at the first stage.

**FIGURE 4:**
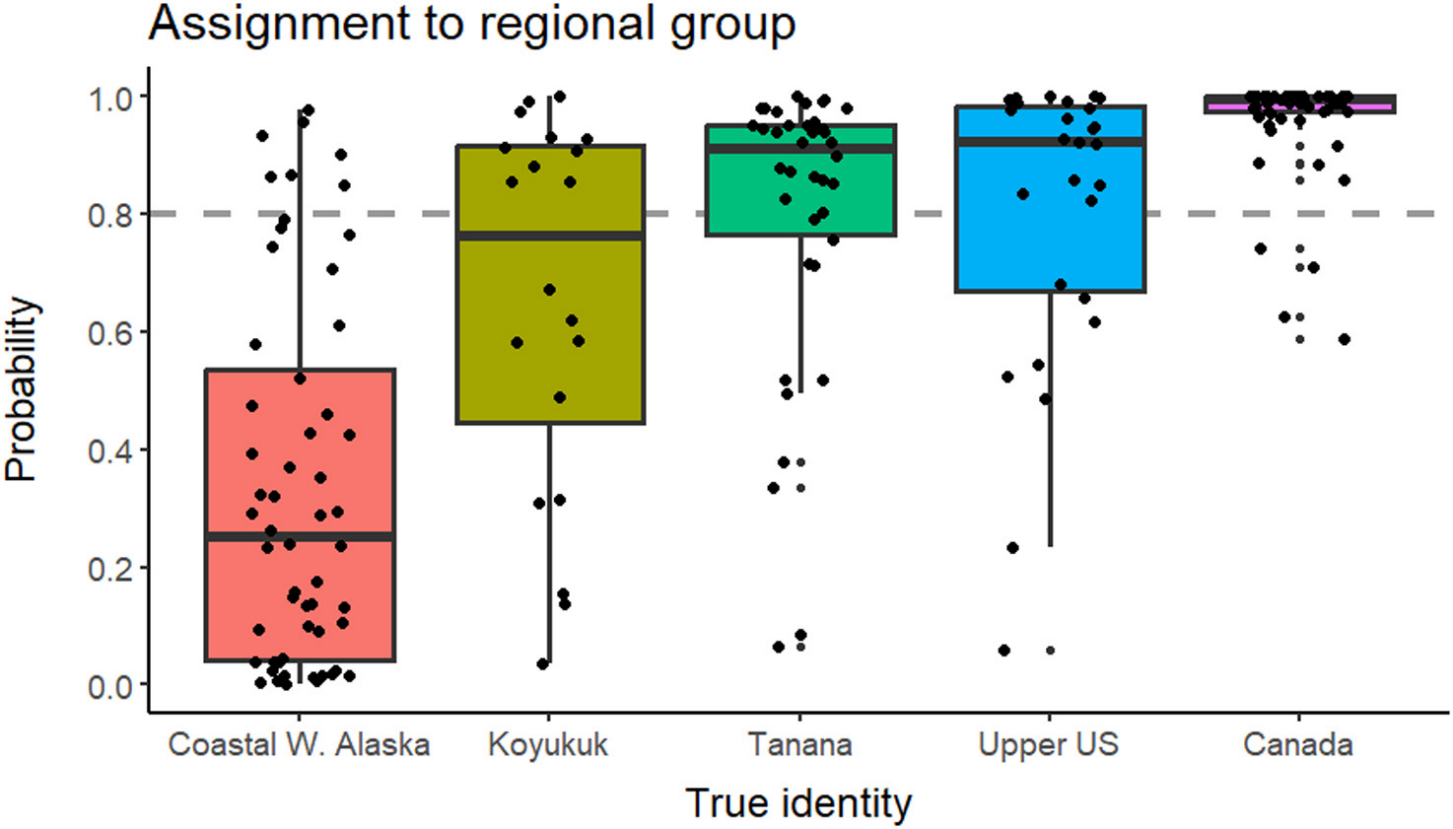
Individual assignment probabilities for assigning fish from each reporting group (*x*‐axis) to the middle/upper Yukon River region during the first stage of the analysis. The true identities of the reporting groups are represented by different colors. Each dot represents an individual fish in the data. The gray‐dashed line indicates the arbitrarily set 0.8 probability threshold for individual reporting group assignment.

Among the regional reporting groups, Koyukuk had the lowest average probability to be assigned to the second stage and was the most likely to be misassigned compared to other Yukon groups (Tanana, Upper US, and Canada; Figure 4).

**FIGURE 5:**
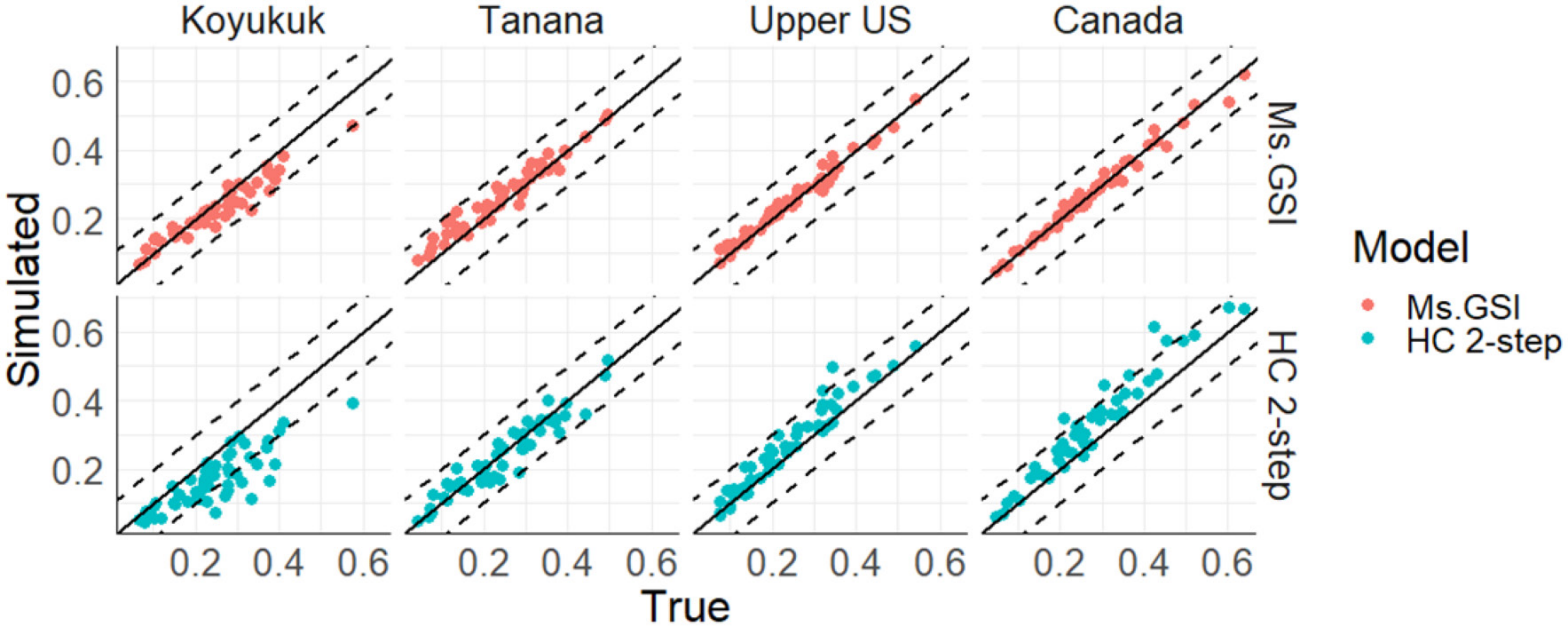
Mean estimated reporting groups proportions are plotted against the true values for each of the 50 cross‐validation analyses. The solid diagonal lines represent where the estimate equals the true value. The dashed lines represent ±0.1 of the true value. All methods use Bering Sea Chinook baseline as the broad‐scale baseline and Yukon River Chinook baseline as the regional baseline. The Coastal Western Alaska group is excluded from this plot because it does not go through the second stage of the analysis for the two‐step approaches.

Model evaluation based on 50 cross‐validation analyses indicated that the Ms.GSI framework improved estimates by lowering deviation, bias, and RMSE compared to a HC 2‐step GSI model (Figure 5; Table 5).

## 4 DISCUSSION

We developed an integrated multistage framework for GSI that can harness the fine‐scale resolution capabilities of regional baselines in the analysis of mixtures containing individuals from a broad geographic range. The Ms.GSI framework allows for disparate baselines to be used in a single integrated process that properly propagates errors from each stage of the analysis. We demonstrated in an example that the Ms.GSI framework can improve accuracy of the estimates, compared to a HC 2‐step GSI approach. This method can be extended to multiple regional baselines within the geographical coverage of the broad‐scale baseline. The Ms.GSI model can use distinct broad‐scale and regional baselines that have no overlap in genetic markers nor population representation as long as the regional reporting groups are within the geographical coverage of the broad‐scale reporting groups. These attributes make this approach much more cost‐effective than past approaches where broad‐scale baselines had to be updated with all critical regionally important markers.

The two‐step analytic procedure was not a new concept. In the past, the two steps were conducted sequentially, and each as a separate process. At the first step of a GSI analysis, everyone in the mixture sample was assigned a membership with a level of certainty. Only individuals with membership assignment probabilities greater than a set threshold (e.g., 0.8) would proceed to the second step. In our cross‐validation analysis, we found that misassignments in the first step made the results at the second step unreliable. In our HC 2‐step example, a 0.8 threshold on the membership assignment probability erroneously included some fish from Coastal W. Alaska while excluding more than half of Koyukuk and a good portion of Upper US and Tanana fish from the second step of the analysis (Figure 4). These misassignments introduced bias in the mixture estimates. Lowering the threshold would include more middle/upper Yukon River fish, but it would risk falsely including more fish from Coastal W. Alaska. Most of all, propagated errors were not accounted for in a HC 2‐step approach and credible intervals were underestimated (Figure 3).

The Ms.GSI model was not impervious to biases either. Although we have shown that the Ms.GSI model had lower RMSE and deviations compared to a HC 2‐step GSI approach, not all biases could be eliminated with the Ms.GSI framework. The Ms.GSI model could not mitigate a well‐known “reporting‐unit bias” (Hasselman et al., 2015; Moran & Anderson, 2018) caused by the variations in the numbers of populations per reporting group when the populations were not well resolved. Moran and Anderson (2018) developed a bootstrap procedure to reduce the reporting‐unit bias and improve accuracy in the GSI estimates, but a similar procedure has not yet been developed for the Ms.GSI framework. Because Ms.GSI requires everyone in the mixture to be genotyped for both the broad‐scale and regional markers, we have not come up with a practical solution to simulate the genotype for individuals that belong to areas outside of the regional baseline.

The Ms.GSI framework provided superior estimates and measurement of uncertainty compared to the conventional HC 2‐step approach. It is arguably more convenient and straightforward to conduct a multi‐baseline GSI analysis in a single process using the Ms.GSI framework (and the Ms.GSI package), as opposed to breaking up the analysis into multiple steps. The Ms.GSI model potentially allows researchers to conduct GSI for a wide geographic area without first developing a large‐scale high‐resolution genetic baseline or dividing mixture samples into smaller regions beforehand. Instead, researchers can utilize existing broad‐scale baselines in combination with regional baselines. A Ms.GSI framework that combines a broad‐scale baseline with regional baselines may make collaborations easier between laboratories, institutions, or agencies from different regions. Most of all, it also means that a high‐resolution baseline would only need to be developed regionally to focus on populations that are difficult to distinguish genetically. The results showed potential for reduction of cost and time in development and improvement of genetic baselines.

## CONFLICT OF INTEREST STATEMENT

There is no conflict of interest.

## Data Availability

Data for this study are available at: to be completed after manuscript is accepted for publication.
